# PBMC proteome is altered in children with high body fat percentage

**DOI:** 10.1038/s41598-025-24461-2

**Published:** 2025-11-19

**Authors:** Maya Petek, Tjaša Hertiš Petek, Uroš Potočnik, Nataša Marčun Varda

**Affiliations:** 1 Faculty of Medicine, University of Maribor, Maribor, Slovenia; 2 Department of Paediatrics, University Medical Centre Maribor, Maribor, Slovenia; 3 Faculty of Chemistry and Chemical Engineering, University of Maribor, Maribor, Slovenia; 4 Department for Science and Research, University Medical Centre Maribor, Maribor, Slovenia

**Keywords:** Paediatric obesity, Proteomics, Peripheral blood mononuclear cells, Body composition, Bioelectrical impedance analysis, Mass spectrometry, Molecular medicine, Proteomics, Obesity, Paediatric research

## Abstract

Obesity in children is an increasing public health issue. Excess adipose tissue, especially in the form of visceral obesity, is associated with poorer cardiometabolic health, persistent low-grade inflammation and oxidative stress. Mass-spectrometry based analysis of peripheral blood mononuclear cells (PBMC) can reveal changes associated with dietary patterns, inflammatory diseases and obesity in adults. We aimed to identify proteomic dysregulations in PBMC of children with obesity, focusing on pathways linked to inflammation and metabolic dysfunction. We isolated cell lysate proteins from blood samples obtained from 71 children and adolescents (aged 5–18) with normal weight, overweight or obesity, measured body composition using bioelectrical impedance analysis, while protein abundances in PBMC lysates were determined using nano-electrospray liquid chromatography coupled with tandem mass spectrometry. Controlling for participant sex, age and leukocyte count, we identified 148 proteins with abundance that was significantly associated with body fat percentage, including protein CutA, several proteins with GTPase activity, and multiple mitochondrial proteins. These obesity-associated changes are better explained by body fat percentage than by height- and weight-based metrics alone, highlighting the utility of body composition analysis for interpreting proteomic results in childhood obesity.

**Supplementary Information:**

The online version contains supplementary material available at 10.1038/s41598-025-24461-2.

## Introduction

Obesity in children and adolescents is a complex chronic disease that affects all body systems and presents a significant global public health challenge^1^. Central or abdominal obesity is especially associated with increased cardiometabolic risk in children and adolescents^2^, as well as with increased blood-bound inflammatory signalling through inflammatory cytokines and adipokines^3^. Multiple approaches are currently used to evaluate obesity, each best suited to specific research and public health goals. Simple anthropometric measures, such as height, weight and body mass index, are easy to obtain, and are widely used for screening and in epidemiological studies but may not give the best insight into individual obesity-related health risks. In contrast, body composition analysis can provide additional information about obesity severity, body fat and muscle mass distribution in an individual, which may better stratify the impact of excessive fat accumulation on health risks^4^.

Body composition can be determined with bioelectrical impedance analysis (BIA), computed tomography (CT), quantitative magnetic resonance imaging (qMRI), dual-energy X-ray absorptiometry (DXA), hydrodensitometry, or anthropometric measurements such as skinfold thickness measurement. DXA scans cause minimal radiation exposure and are commonly used to assess body composition in adults^5^ but are challenging in paediatric populations, as they take > 10 min to complete and require the participant to lie perfectly still. The gold standard method (CT) is considered inappropriate for research use in children because of significant radiation exposure^6^. Bioelectrical impedance analysis is easily accessible, affordable, and free from radiation use, which makes it suitable for use in hospitals, fitness centres, and even at home^7^. BIA measurement of body composition is a well-characterised and validated method in adult populations and in children. As a non-invasive method suitable for bedside use, BIA body composition measurement is particularly valuable in children, where minimally invasive investigations are strongly preferred, for example for assessing nutritional status and growth^7,8^. Consequently, BIA has become the most widely used method for assessing body composition^9^ in part due to BIA-based estimates of body fat percentage (BFP) showing better reproducibility than alternative non-invasive methods^10^.

Peripheral blood mononuclear cells (PBMC) are a readily accessible blood fraction isolated from whole blood using density gradient centrifugation. They are composed primarily of T-lymphocytes (approximately 70%), B-lymphocytes (15%), natural killer cells (10%), monocytes (5%) and dendritic cells. In the absence of an ongoing immune response, most PBMC are naïve or resting cells without active effector functions^11^. Upon activation, PBMC are key drivers of both normal and pathological immune responses, which is reflected in the protein expression patterns in activated cells. Changes in protein expression patterns in PBMC have been observed in multiple cancers^12,13^, long COVID^14^, multiple sclerosis^15^, rheumatoid arthritis^16^, psychiatric conditions^17,18^ and other diseases^19^. Gene and protein expression profiles in peripheral blood cells can reflect whole body adaptation status from metabolic and physio-pathological states, which makes PBMC a promising source of biomarkers in nutrigenomic and metabolic studies^20^.

Here we present a new mass spectrometry-based analysis of the PBMC proteome in a previously described cohort of children and adolescents with normal weight, overweight or obesity^21^. Published results show that in this paediatric cohort, overweight or obesity (measured by body mass index relative to reference growth charts) was associated with increased blood leukocytes, higher systolic and diastolic blood pressure, lower high-density lipoprotein, higher myeloperoxidase and lower vitamin D levels^22^. Here, we isolated PBMCs from participant blood samples and used liquid chromatography coupled with tandem mass spectrometry to obtain proteome profiles of abundant circulating immune cells. Our aim was to investigate whether the PBMC proteome shows an altered state depending on obesity status in children, and if we can identify protein molecular signatures that are associated with unfavourable body composition.

## Results

### Characteristics of the study population

Participants in this work were drawn from a population enrolled in a previous study, which has been described in detail^21,22^. Participant body composition was assessed using bioelectrical impedance analysis (BIA). Compared to the original cohort, we excluded participants whose body composition was not measured using BIA or whose PBMC protein samples were of insufficient quality. Characteristics of the study population investigated in this proteomics study are summarised in Table 1; it comprised 71 children and adolescents, 31 (44%) of which were girls. Participant ages ranged 5–18 years of age at the time of study participation, with mean age 13.7 and median age 14 years old (interquartile range 12.5 to 16.0 years old). Twenty-eight children (12 female, 43%) presented with a body mass index (BMI) within 5-85th percentile according to CDC growth charts^23^ and were considered normal weight, 4 children (3 female) were in the overweight range above the 85th percentile, and 39 (16 female, 41%) were considered obese with BMI measuring above 95th percentile for their age and sex.

**Table 1:** Clinical characteristics of the participant cohort.

	Normal weight	Overweight & obese	P value
N (% female)	28 (43%)	43 (44%)	1.00
Age (years)	14.0 (3.3)	14.0 (3.0)	0.37
Height (cm)	165 (17)	169 (16)	0.41
Weight (kg)	56.5 (18.1)	88.0 (35.9)	< 0.0001
Percentile BMI	57.9 (36.1)	98.3 (2.25)	< 0.0001
Waist (cm)	70 (6.3)	94 (19)	< 0.0001
Hip (cm)	85 (9.3)	109 (17)	< 0.0001
Subcutaneous fat (mm)	12 (7)	36 (16)	< 0.0001
Visceral fat (mm)	39 (11)	60 (25)	< 0.0001
Body fat percentage (%)	21.4% (8.3%)	34.7% (7.8%)	< 0.0001
Fat mass (FM) (kg)	11.3 (5.9)	30.7 (13.4)	< 0.0001
Fat-free mass (FFM) (kg)	44.2 (10.4)	57.7 (22.1)	< 0.0001
Body cell mass (BCM) (kg)	23.4 (6.0)	31.6 (14.4)	< 0.001
Extracellular water (ECW) (L)	14.2 (3.3)	17.9 (6.5)	< 0.001
Total body water (TBW) (L)	32.6 (6.9)	42.6 (14.3)	< 0.0001
Phase angle (°)	6.1 (1.0)	6.4 (0.8)	0.080
Leukocytes (× 10^9^/L)	5.72 (1.87)	6.94 (3.67)	0.022

Selected demographic characteristics, body measurements and body composition measurements of the participants are presented grouped by BMI status. Unless otherwise specified, values in the normal weight and overweight/obese columns are shown as median (interquartile range). P values for differences between groups were calculated as follows: Sex distribution, chi-squared test; all other parameters: Mann Whitney U test.

**Fig. 1:**
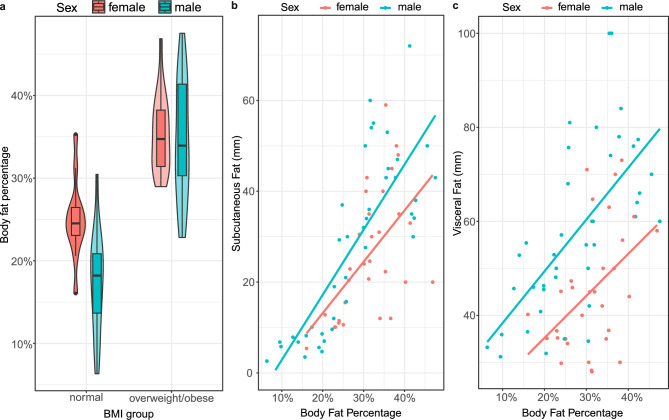
Correlations between body fat percentage (BFP) and (**a**) BMI group, (**b**) subcutaneous and (**c**) visceral fat thickness. Participants in the normal weight BMI group have a lower BFP than participants with overweight or obesity. In the normal weight group, female participants have a higher BFP, but the difference is diminished in the group with overweight or obesity. BFP correlates well with abdominal subcutaneous and visceral fat thickness in both genders.

At inclusion, body composition measurement was done using BIA (Table 1), which generated six parameters including total fat mass and total body mass. We calculated the body fat percentage (BFP) by dividing BIA-estimated total fat mass with total body mass. Both male and female participants have a significantly higher BFP in the overweight/obese group (Fig. 1a). Additionally, we observe a difference in the BFP distribution between female and male participants. In the normal weight group, girls have a higher BFP compared to boys (25% vs 17%, p < 0.001, Mann Whitney U test), reflecting healthy development^24^. However, in the overweight/obese group, there is no significant difference in BFP between male and female participants (35% vs 35%, p = 0.78). Children with overweight/obesity also have increased leukocyte counts compared to the normal weight group (Sup. Figs.  1, 2). Finally, ultrasound examination was used to measure subcutaneous and visceral fat thickness in the abdomen, which provide a measure of central obesity (Fig. 1). These measurements show that boys have more visceral fat at equivalent overall BFP, and that BFP correlates well with both subcutaneous and visceral fat thickness in the abdomen.

**Fig. 2:**
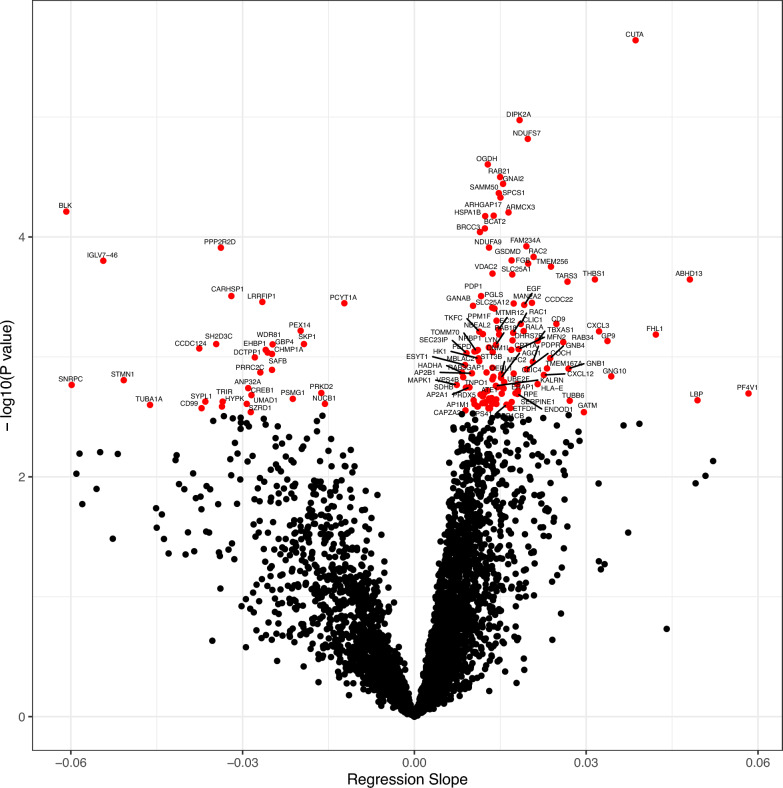
Protein differential expression as a function of BFP. Horizonal axis shows the slope of each correlation coefficient (effect size), derived from protein abundance on a logarithmic scale and BFP expressed as a percentage. Vertical axis shows decimal logarithms of raw *P* values. Proteins with significantly correlating expression (*q*-value < 0.05) are shown in red and labelled with corresponding gene names. Proteins upregulated in participants with higher BFP are on the right-hand side.

### Differential protein abundance in the PBMC proteome

We obtained a mass spectrometry PBMC proteome dataset from 71 participants, which contained 4068 proteins that were quantified in at least 50% of samples. We explored differential protein abundance as a function of body fat percentage using *limma* to perform linear modelling. BIA-assessed body fat percentage was set as the key explanatory variable of interest and leukocyte counts, participant age, participant sex and sample batch were added as covariates. Leukocyte counts were included as a covariate because they are mildly but significantly elevated in the overweight/obese group, which may affect PBMC proteome composition in particular (Table 1). We observed that the abundance of 148 proteins significantly correlated with BFP (Fig. 2, Table 2, Sup. Tab. S1). Heatmap visualisation shows that protein abundance changes are not driven by individual participants but reflect overall tendencies (Sup. Fig. S3).

**Table 2:** Proteins with significant differential abundance as a function of body fat percentage.

Accession	Gene	Protein name	Average expression	logFC	q-value
Abundance positively correlated with body fat percentage					
O60888	CUTA	Protein CutA	22.61	0.0386	0.006
Q8NDZ4	DIPK2A	Divergent protein kinase domain 2A	20.78	0.0184	0.013
O75251	NDUFS7	NADH dehydrogenase [ubiquinone] iron-sulfur protein 7, mitochondrial	20.43	0.0198	0.013
Q02218	OGDH	2-oxoglutarate dehydrogenase complex component E1	20.76	0.0128	0.014
Q9UL25	RAB21	Ras-related protein Rab-21	22.34	0.0150	0.014
P04899	GNAI2	Guanine nucleotide-binding protein G(i) subunit alpha-2	23.36	0.0155	0.014
Q9Y512	SAMM50	Sorting and assembly machinery component 50 homolog	21.19	0.0147	0.014
Q9Y6A9	SPCS1	Signal peptidase complex subunit 1	22.52	0.0150	0.014
Q9UH62	ARMCX3	Armadillo repeat-containing X-linked protein 3	20.69	0.0164	0.014
Q68EM7	ARHGAP17	Rho GTPase-activating protein 17	20.64	0.0139	0.014
P0DMV9	HSPA1B	Heat shock 70 kDa protein 1B	22.72	0.0124	0.014
O15382	BCAT2	Branched-chain-amino-acid aminotransferase, mitochondrial	20.50	0.0123	0.017
P46736	BRCC3	Lys-63-specific deubiquitinase BRCC36	20.64	0.0115	0.017
Q9H0X4	FAM234A	Protein FAM234A	21.52	0.0196	0.019
Q16795	NDUFA9	NADH dehydrogenase [ubiquinone] 1 alpha subcomplex subunit 9, mitochondrial	21.75	0.0130	0.019
P15153	RAC2	Ras-related C3 botulinum toxin substrate 2	23.81	0.0208	0.020
P57764	GSDMD	Gasdermin-D	20.22	0.0170	0.020
P02675	FGB	Fibrinogen beta chain	24.58	0.0199	0.020
Q8N2U0	TMEM256	Transmembrane protein 256	21.24	0.0239	0.021
P45880	VDAC2	Voltage-dependent anion-selective channel protein 2	23.34	0.0136	0.022
P53007	SLC25A1	Tricarboxylate transport protein, mitochondrial	21.32	0.0171	0.022
P07996	THBS1	Thrombospondin-1	24.05	0.0315	0.022
Q7L211	ABHD13	Protein ABHD13	17.81	0.0481	0.022
A2RTX5	TARS3	Threonine–tRNA ligase 2, cytoplasmic	20.37	0.0267	0.022
Q9P0J1	PDP1	[Pyruvate dehydrogenase [acetyl-transferring]]-phosphatase 1, mitochondrial	20.23	0.0117	0.027
O60826	CCDC22	Coiled-coil domain-containing protein 22	20.10	0.0205	0.027
P49641	MAN2A2	Alpha-mannosidase 2x	20.29	0.0173	0.027
P01133	EGF	Pro-epidermal growth factor	20.82	0.0192	0.027
Q14697	GANAB	Neutral alpha-glucosidase AB	22.34	0.0102	0.027
O75746	SLC25A12	Electrogenic aspartate/glutamate antiporter SLC25A12, mitochondrial	20.39	0.0136	0.027
O95336	PGLS	6-phosphogluconolactonase	22.67	0.0140	0.027
P49593	PPM1F	Protein phosphatase 1F	21.20	0.0143	0.034
P21926	CD9	CD9 antigen	25.18	0.0248	0.034
P63000	RAC1	Ras-related C3 botulinum toxin substrate 1	23.22	0.0186	0.034
Q9C0I1	MTMR12	Myotubularin-related protein 12	20.83	0.0147	0.034
P11233	RALA	Ras-related protein Ral-A	22.33	0.0191	0.034
P19876	CXCL3	C-X-C motif chemokine 3	20.32	0.0323	0.034
Q3LXA3	TKFC	Triokinase/FMN cyclase	21.51	0.0113	0.034
O00299	CLIC1	Chloride intracellular channel protein 1	24.15	0.0173	0.034
Q6ZNJ1	NBEAL2	Neurobeachin-like protein 2	21.23	0.0120	0.034
O75521	ECI2	Enoyl-CoA delta isomerase 2	22.44	0.0148	0.034
Q13642	FHL1	Four and a half LIM domains protein 1	24.30	0.0422	0.034
Q6IAN0	DHRS7B	Dehydrogenase/reductase SDR family member 7B	20.33	0.0171	0.035
P24557	TBXAS1	Thromboxane-A synthase	22.57	0.0215	0.035
P14770	GP9	Platelet glycoprotein IX	24.75	0.0337	0.035
Q9BZG1	RAB34	Ras-related protein Rab-34	19.38	0.0260	0.035
Q9NP72	RAB18	Ras-related protein Rab-18	22.24	0.0142	0.035
P07948	LYN	Tyrosine-protein kinase Lyn	22.01	0.0130	0.035
O95140	MFN2	Mitofusin-2	20.75	0.0182	0.035
P50416	CPT1A	Carnitine O-palmitoyltransferase 1, liver isoform	21.22	0.0169	0.035
Q9UHY1	NRBP1	Nuclear receptor-binding protein	21.12	0.0111	0.035
O94826	TOMM70	Mitochondrial import receptor subunit TOM70	21.38	0.0108	0.035
P12955	PEPD	Xaa-Pro dipeptidase	21.11	0.0105	0.035
O00429	DNM1L	Dynamin-1-like protein	22.74	0.0137	0.035
Q9Y6Y8	SEC23IP	SEC23-interacting protein	20.30	0.0091	0.035
Q9HAV0	GNB4	Guanine nucleotide-binding protein subunit beta-4	21.50	0.0237	0.037
P19367	HK1	Hexokinase-1	23.27	0.0112	0.037
Q68D91	MBLAC2	Acyl-coenzyme A thioesterase MBLAC2	20.51	0.0113	0.038
Q8NCN5	PDPR	Pyruvate dehydrogenase phosphatase regulatory subunit, mitochondrial	19.82	0.0207	0.038
Q9BSJ8	ESYT1	Extended synaptotagmin-1	22.08	0.0088	0.040
Q8TCJ2	STT3B	Dolichyl-diphosphooligosaccharide–protein glycosyltransferase subunit STT3B	21.49	0.0136	0.041
Q8TBQ9	TMEM167A	Protein kish-A	21.53	0.0232	0.041
P62873	GNB1	Guanine nucleotide-binding protein G(I)/G(S)/G(T) subunit beta-1	23.28	0.0269	0.041
O43405	COCH	Cochlin	20.49	0.0196	0.041
Q15042	RAB3GAP1	Rab3 GTPase-activating protein catalytic subunit	20.15	0.0126	0.041
P40939	HADHA	Trifunctional enzyme subunit alpha, mitochondrial	22.28	0.0083	0.041
P63010	AP2B1	AP-2 complex subunit beta	21.26	0.0100	0.041
Q9Y696	CLIC4	Chloride intracellular channel protein 4	22.32	0.0173	0.041
O95563	MPC2	Mitochondrial pyruvate carrier 2	21.24	0.0152	0.042
P48061	CXCL12	Stromal cell-derived factor 1	20.82	0.0226	0.042
P50151	GNG10	Guanine nucleotide-binding protein G(I)/G(S)/G(O) subunit gamma-10	20.29	0.0344	0.042
Q9BUN8	DERL1	Derlin-1	21.69	0.0138	0.042
O75351	VPS4B	Vacuolar protein sorting-associated protein 4B	21.62	0.0085	0.042
Q9UL18	AGO1	Protein argonaute-1	20.11	0.0151	0.042
Q92973	TNPO1	Transportin-1	20.45	0.0136	0.043
Q969M7	UBE2F	NEDD8-conjugating enzyme UBE2F	21.01	0.0157	0.044
P13747	HLA-E	HLA class I histocompatibility antigen, alpha chain E	21.23	0.0215	0.045
Q9NZ08	ERAP1	Endoplasmic reticulum aminopeptidase 1	21.84	0.0180	0.045
P28482	MAPK1	Mitogen-activated protein kinase 1	22.02	0.0074	0.045
O60229	KALRN	Kalirin	21.43	0.0142	0.045
Q9BZH6	WDR11	WD repeat-containing protein 11	20.67	0.0145	0.045
O95782	AP2A1	AP-2 complex subunit alpha-1	21.32	0.0096	0.045
Q86UT6	NLRX1	NLR family member X1	20.65	0.0154	0.045
P21912	SDHB	Succinate dehydrogenase [ubiquinone] iron-sulfur subunit, mitochondrial	21.53	0.0090	0.045
P51790	CLCN3	H( +)/Cl(-) exchange transporter 3	19.97	0.0156	0.045
Q96A26	FAM162A	Protein FAM162A	22.82	0.0128	0.046
Q96AT9	RPE	Ribulose-phosphate 3-epimerase	21.83	0.0181	0.046
O95260	ATE1	Arginyl-tRNA–protein transferase 1	20.37	0.0122	0.046
P05121	SERPINE1	Plasminogen activator inhibitor 1	22.18	0.0177	0.046
P10720	PF4V1	Platelet factor 4 variant	23.91	0.0584	0.046
Q8NBX0	SCCPDH	Saccharopine dehydrogenase-like oxidoreductase	21.25	0.0153	0.046
O94919	ENDOD1	Endonuclease domain-containing 1 protein	22.78	0.0180	0.046
P30044	PRDX5	Peroxiredoxin-5, mitochondrial	22.51	0.0116	0.046
O43615	TIMM44	Mitochondrial import inner membrane translocase subunit TIM44	20.49	0.0120	0.047
Q9H7D0	DOCK5	Dedicator of cytokinesis protein 5	20.35	0.0135	0.047
Q9NR19	ACSS2	Acetyl-coenzyme A synthetase, cytoplasmic	20.61	0.0130	0.047
Q9UHQ9	CYB5R1	NADH-cytochrome b5 reductase 1	21.50	0.0143	0.047
P18428	LBP	Lipopolysaccharide-binding protein	20.29	0.0494	0.047
Q9BXS5	AP1M1	AP-1 complex subunit mu-1	22.07	0.0103	0.047
Q9BUF5	TUBB6	Tubulin beta-6 chain	20.95	0.0271	0.047
P61225	RAP2B	Ras-related protein Rap-2b	22.25	0.0134	0.047
Q16134	ETFDH	Electron transfer flavoprotein-ubiquinone oxidoreductase, mitochondrial	19.84	0.0170	0.047
O75131	CPNE3	Copine-3	21.91	0.0125	0.047
P61106	RAB14	Ras-related protein Rab-14	23.19	0.0119	0.047
Q13496	MTM1	Myotubularin	20.41	0.0124	0.047
P27348	YWHAQ	14–3-3 protein theta	22.42	0.0136	0.047
Q16762	TST	Thiosulfate sulfurtransferase	22.43	0.0143	0.047
Q969X5	ERGIC1	Endoplasmic reticulum-Golgi intermediate compartment protein 1	21.24	0.0105	0.047
P49754	VPS41	Vacuolar protein sorting-associated protein 41 homolog	19.75	0.0161	0.047
O15173	PGRMC2	Membrane-associated progesterone receptor component 2	22.02	0.0111	0.047
Q9Y678	COPG1	Coatomer subunit gamma-1	21.40	0.0110	0.047
P25325	MPST	3-mercaptopyruvate sulfurtransferase	23.34	0.0130	0.047
P62140	PPP1CB	Serine/threonine-protein phosphatase PP1-beta catalytic subunit	21.91	0.0168	0.047
P11182	DBT	Lipoamide acyltransferase component of branched-chain alpha-keto acid dehydrogenase complex, mitochondrial	20.40	0.0129	0.047
Q8N5M4	TTC9C	Tetratricopeptide repeat protein 9C	20.84	0.0132	0.047
P47755	CAPZA2	F-actin-capping protein subunit alpha-2	23.50	0.0089	0.049
P50440	GATM	Glycine amidinotransferase, mitochondrial	20.20	0.0296	0.050
Abundance negatively correlated with body fat percentage					
P51451	BLK	Tyrosine-protein kinase Blk	19.05	-0.0608	0.014
Q66LE6	PPP2R2D	Serine/threonine-protein phosphatase 2A 55 kDa regulatory subunit B delta isoform	19.99	-0.0338	0.019
A0A075B6I9	IGLV7-46	Immunoglobulin lambda variable 7–46	18.32	-0.0543	0.020
Q9Y2V2	CARHSP1	Calcium-regulated heat-stable protein 1	20.55	-0.0320	0.027
Q32MZ4	LRRFIP1	Leucine-rich repeat flightless-interacting protein 1	20.80	-0.0266	0.027
P49585	PCYT1A	Choline-phosphate cytidylyltransferase A	21.02	-0.0122	0.027
O75381	PEX14	Peroxisomal membrane protein PEX14	19.12	-0.0199	0.034
P63208	SKP1	S-phase kinase-associated protein 1	20.60	-0.0193	0.035
Q8N5H7	SH2D3C	SH2 domain-containing protein 3C	18.23	-0.0346	0.035
Q562E7	WDR81	WD repeat-containing protein 81	18.30	-0.0248	0.035
Q96CT7	CCDC124	Coiled-coil domain-containing protein 124	18.61	-0.0376	0.035
Q8NDI1	EHBP1	EH domain-binding protein 1	19.35	-0.0260	0.035
Q96PP9	GBP4	Guanylate-binding protein 4	19.93	-0.0256	0.035
Q9HD42	CHMP1A	Charged multivesicular body protein 1a	20.71	-0.0249	0.035
Q9H773	DCTPP1	dCTP pyrophosphatase 1	18.61	-0.0279	0.037
Q15424	SAFB	Scaffold attachment factor B1	19.55	-0.0249	0.041
Q9Y520	PRRC2C	Protein PRRC2C	18.62	-0.0269	0.041
P16949	STMN1	Stathmin	19.28	-0.0508	0.043
P09234	SNRPC	U1 small nuclear ribonucleoprotein C	19.81	-0.0599	0.045
P39687	ANP32A	Acidic leucine-rich nuclear phosphoprotein 32 family member A	20.58	-0.0290	0.045
Q9BZL6	PRKD2	Serine/threonine-protein kinase D2	20.28	-0.0163	0.046
P16220	CREB1	Cyclic AMP-responsive element-binding protein 1	20.24	-0.0285	0.046
O95456	PSMG1	Proteasome assembly chaperone 1	19.08	-0.0212	0.047
Q16563	SYPL1	Synaptophysin-like protein 1	21.14	-0.0365	0.047
Q9BQ61	TRIR	Telomerase RNA component interacting RNase	19.28	-0.0335	0.047
Q02818	NUCB1	Nucleobindin-1	21.01	-0.0156	0.047
C9J7I0	UMAD1	UBAP1-MVB12-associated (UMA)-domain containing protein 1	19.33	-0.0293	0.047
Q71U36	TUBA1A	Tubulin alpha-1A chain	20.95	-0.0462	0.047
Q9NX55	HYPK	Huntingtin-interacting protein K	19.50	−0.0336	0.047
P14209	CD99	CD99 antigen	19.82	-0.0372	0.047
Q7Z422	SZRD1	SUZ domain-containing protein 1	20.79	-0.0286	0.05

Proteins are grouped by direction of correlation and ordered by increasing q-value. Average Expression denotes the average protein intensity on a log2 scale. LogFC = log2-fold change in protein abundance per unit change in BFP, ie. logF.

In total, 117 proteins show increased abundance in correlation with increased BFP. These include protein CutA, two chemokines, C-X-C motif chemokine 3 (CXCL3) and stromal cell-derived factor 1 (SDF1, gene *CXCL12*), and tyrosine-protein kinase Lyn (LYN), a key player in B-cell activation after B-cell receptor crosslinking. Thirty-one proteins decrease in PBMC abundance in participants with increased BFP, amongst them are B-lymphoid tyrosine kinase (BLK), the delta isoform of the 55 kDa regulatory subunit B of protein phosphatase 2A (2ABD), peroxisomal membrane protein PEX14 and WD repeat-containing protein 81 (WDR81).

### Gene set enrichment analysis

**Fig. 3:**
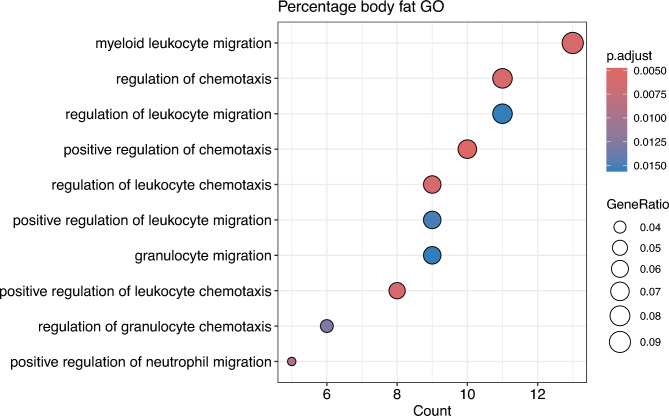
GO analysis of proteins significantly correlated with BFP in patients. Ontologies for cellular component, molecular function and biological process were evaluated simultaneously.

To better understand the functional role of differentially expressed proteins, we performed Gene Ontology (GO) term enrichment analysis. This approach compares GO annotations for cellular components, molecular function and biological processes between the proteins present in the differentially expressed protein set against the set of annotations linked with all observed proteins. We examined whether the proteins significantly changing with BFP were enriched any of these three ontologies in comparison to all identified proteins (Table 3). In the cellular components ontology, three terms were significant: mitochondrial membrane (GO:0,031,966) and mitochondrial envelope (GO:0,005,740), both with the same 24 proteins. The second enriched cellular component was extrinsic component of cytoplasmic side of plasma membrane (GO:0,031,234), which was associated with 7 proteins, including GNAI2, BLK and LYN. Most significant GO terms were identified in the biological processes ontology, which included GO terms for myeloid leukocyte migration, (positive) regulation of chemotaxis, and regulation of leukocyte migration more generally. Granulocyte and neutrophil migration are also amongst GO enriched terms (Fig. 3). Several of these GO terms included the proteins RAC2, RAC2, TSP1, CXCL3, DNM1L, SDF1, LBP, MAPK1 and others (Table 3).

**Table 3:** Gene ontology enrichment results for the combined analysis in all three ontologies.

Ontology	GO term ID	Description	Gene ratio	Background ratio	P value	q value	geneID
CC	GO:0,031,966	Mitochondrial membrane	24/143	296/3978	0.000107	0.0276	NDUFS7, OGDH, SAMM50, ARMCX3, NDUFA9, RAC2, GSDMD, VDAC2, SLC25A1, SLC25A12, LYN, MFN2, CPT1A, TOMM70, DNM1L, HK1, HADHA, MPC2, NLRX1, SDHB, FAM162A, TIMM44, ETFDH, GATM
CC	GO:0,031,234	Extrinsic component of cytoplasmic side of plasma membrane	7/143	35/3978	0.000193	0.0276	GNAI2, BLK, LYN, GNB4, ESYT1, GNB1, GNG10
CC	GO:0,005,740	Mitochondrial envelope	24/143	318/3978	0.000324	0.0308	NDUFS7, OGDH, SAMM50, ARMCX3, NDUFA9, RAC2, GSDMD, VDAC2, SLC25A1, SLC25A12, LYN, MFN2, CPT1A, TOMM70, DNM1L, HK1, HADHA, MPC2, NLRX1, SDHB, FAM162A, TIMM44, ETFDH, GATM
BP	GO:0,050,921	Positive regulation of chemotaxis	10/142	43/3893	0.000002	0.0046	RAC2, THBS1, PPM1F, RAC1, DNM1L, CXCL12, MAPK1, PRKD2, SERPINE1, LBP
BP	GO:0,097,529	Myeloid leukocyte migration	13/142	81/3893	0.000005	0.0056	RAC2, THBS1, CD9, RAC1, CXCL3, LYN, DNM1L, CXCL12, MAPK1, SERPINE1, PF4V1, LBP, CD99
BP	GO:0,002,688	Regulation of leukocyte chemotaxis	9/142	40/3893	0.000009	0.0056	RAC2, THBS1, RAC1, LYN, DNM1L, CXCL12, MAPK1, SERPINE1, LBP
BP	GO:0,002,690	Positive regulation of leukocyte chemotaxis	8/142	31/3893	0.000010	0.0056	RAC2, THBS1, RAC1, DNM1L, CXCL12, MAPK1, SERPINE1, LBP
BP	GO:0,050,920	Regulation of chemotaxis	11/142	63/3893	0.000012	0.0056	RAC2, THBS1, PPM1F, RAC1, LYN, DNM1L, CXCL12, MAPK1, PRKD2, SERPINE1, LBP
BP	GO:1,902,624	Positive regulation of neutrophil migration	5/142	11/3893	0.000023	0.0087	RAC2, RAC1, DNM1L, LBP, CD99
BP	GO:0,071,622	Regulation of granulocyte chemotaxis	6/142	19/3893	0.000039	0.0124	RAC2, THBS1, RAC1, DNM1L, MAPK1, LBP
BP	GO:0,002,687	Positive regulation of leukocyte migration	9/142	49/3893	0.000052	0.0146	RAC2, THBS1, RAC1, DNM1L, CXCL12, MAPK1, SERPINE1, LBP, CD99
BP	GO:0,097,530	Granulocyte migration	9/142	50/3893	0.000062	0.0152	RAC2, THBS1, RAC1, CXCL3, DNM1L, MAPK1, PF4V1, LBP, CD99
BP	GO:0,002,685	Regulation of leukocyte migration	11/142	75/3893	0.000068	0.0152	RAC2, THBS1, CD9, RAC1, LYN, DNM1L, CXCL12, MAPK1, SERPINE1, LBP, CD99
BP	GO:0,030,595	Leukocyte chemotaxis	11/142	78/3893	0.000098	0.0197	RAC2, THBS1, RAC1, CXCL3, LYN, DNM1L, CXCL12, MAPK1, SERPINE1, PF4V1, LBP
BP	GO:0,071,621	Granulocyte chemotaxis	8/142	42/3893	0.000106	0.0197	RAC2, THBS1, RAC1, CXCL3, DNM1L, MAPK1, PF4V1, LBP
BP	GO:1,902,622	Regulation of neutrophil migration	5/142	15/3893	0.000135	0.0224	RAC2, RAC1, DNM1L, LBP, CD99
BP	GO:0,060,326	Cell chemotaxis	12/142	95/3893	0.000141	0.0224	RAC2, THBS1, RAC1, CXCL3, LYN, DNM1L, CXCL12, MAPK1, PRKD2, SERPINE1, PF4V1, LBP
BP	GO:0,071,624	Positive regulation of granulocyte chemotaxis	4/142	10/3893	0.000300	0.0380	RAC2, RAC1, DNM1L, LBP
BP	GO:0,090,023	Positive regulation of neutrophil chemotaxis	4/142	10/3893	0.000300	0.0380	RAC2, RAC1, DNM1L, LBP
BP	GO:0,006,935	Chemotaxis	14/142	134/3893	0.000307	0.0380	RAC2, THBS1, PPM1F, RAC1, RALA, CXCL3, LYN, DNM1L, CXCL12, MAPK1, PRKD2, SERPINE1, PF4V1, LBP
BP	GO:0,042,330	Taxis	14/142	134/3893	0.000307	0.0380	RAC2, THBS1, PPM1F, RAC1, RALA, CXCL3, LYN, DNM1L, CXCL12, MAPK1, PRKD2, SERPINE1, PF4V1, LBP

Results include up- and down-regulated proteins analysed simultaneously. Ontologies: BP, biological process; CC, cellular component; MF, molecular function. Only significant GO terms are shown, filtered for q-value < 0.05 after multiple testing correction.

Finally, we performed separate GO term enrichment analyses for up- and down-regulated proteins. The analysis of down-regulated proteins did not reveal any significantly associated GO terms, likely due to the lower number of proteins in this group. When we explored the GO enrichment of the 117 significantly more abundant proteins (Sup. Tab. S2), we observed an additional significant result in the molecular function ontology: ‘GTPase activity’ (GO:0,003,924) was significant and linked with 14 proteins: RAB21, GNAI2, RAC2, RAC1, RALA, RAB34, RAB18, MFN2, DNM1L, GNB1, GNG10, TUBB6, RAP2B and RAB14. In the cellular component ontology, twenty-one GO terms were enriched (Sup. Fig.  4), relating to the mitochondrial membrane and mitochondrial matrix, heterotrimeric G-protein complex, coated vesicles and vesicle membrane. Finally, the biological processes GO analysis showed largely the same terms (Sup. Fig.  5) as the overall GO analysis for all significant proteins.

## Discussion

Previous work with the paediatric cohort in this study explored correlations between adiposity indices and individual markers of inflammation and oxidative stress^21,22^. Children with overweight or obesity measured by BMI were more likely to have lower vitamin D level, increased leukocyte counts and C-reactive protein levels, increased blood triglycerides and decreased high density lipoprotein values, compared to the normal weight group. Children with increased BMI also showed increased levels of serum myeloperoxidase, a marker of prolonged low-grade inflammation that results from neutrophil infiltration in adipose tissue^25^, and reduced levels of adiponectin, a protective adipokine involved in the control of fat metabolism and insulin sensitivity. However, published work did not investigate wider proteome expression in any blood or tissue sample. Here, we extended the analysis with an investigation of how the PBMC proteome is altered depending on obesity status in young participants aged 5–18, and whether protein molecular signatures could be identified that are associated with unfavourable body composition. We found that the PBMC proteome profile is statistically significantly altered in a paediatric population with obesity and increased body fat percentage, and the association remains significant after controlling for participant age, sex and leukocyte counts.

We observed that a higher BFP was associated with significant differences in proteome abundance profiles. One hundred and seventeen proteins show statistically significant increased expression in participants with higher BFP. The protein with the lowest q-value (0.016) after multiple testing correction was protein CutA (CUTA), which was upregulated in participants with higher BFP. CUTA is a 19 kDa membrane protein that may form a part of protein complex attached to acetylcholinesterase but whose function is still unclear. The CutA protein family is widely distributed across prokaryotes and eukaryote organism and CUTA likely acts as a transporter of unknown cargo^26^. The function of this protein in humans is unclear and somewhat controversial: it has been identified as a substrate of matrix metalloproteinase 13^27^, and if present in sweat, proposed as a biomarker of cystic fibrosis^28^. CUTA involvement has been proposed in mediating acetylcholinesterase activity, copper homeostasis, and in regulating cleavage of β-amyloid precursor protein^29^.

To further interpret our results, we performed gene set enrichment analyses using the Gene Ontology database. First, we explored three subsets of GO enrichment for all 148 significantly different proteins together. Most significant GO terms were found in the biological process ontology, describing myeloid and granulocyte leukocyte migration, chemotaxis and regulation of these processes. These GO terms strongly featured Ras-related C3 botulinum toxin substrate 2 (RAC2), Ras-related C3 botulinum toxin substrate 1 (RAC1), thrombospondin 1 (TSP1), protein phosphatase 1F (PPM1F), dynamin-1-like protein (DNM1L), mitogen-activated protein kinase 1 (MAPK1) and lipopolysaccharide-binding protein (LBP). RAC1 and RAC2 are Rho-GTPases whose activity is regulated by switching between binding GTP and GDP, and their activity is crucial for regulating different aspects of cell migration. While RAC1 is widely expressed, RAC2 is restricted to the hematopoietic system^30^. Interestingly, CUTA was not associated with any significant GO terms.

The cellular compartments GO analysis had three significant terms, two of which were mitochondrial membrane and mitochondrial envelope, each with 24 proteins. The most significant of these 24 is NADH dehydrogenase [ubiquinone] iron-sulfur protein 7 (NDUFS7), the core subunit of respiratory Complex I in mitochondria and a key source of reactive oxygen species, especially superoxide^31^. Increased oxidative stress is well established in obesity and has been previously demonstrated in the present paediatric cohort, especially in boys ^21^. The third significant cellular compartments term, extrinsic component of cytoplasmic side of plasma membrane, includes GNAI2 which interacts with other adipose tissue-specific proteins, and non-receptor tyrosine kinases BLK (downregulated) and LYN (upregulated). LYN regulates B-cell development by phosphorylation of immunoreceptor tyrosine-based inhibitory motifs, which then lead to signal transduction modulation.

We further explored separate GO term enrichment for down- and up-regulated proteins. We did not see significant GO term enrichment in the downregulated proteins, possibly because fewer proteins have reduced abundance in participants with higher BFP. Individually, the most significant downregulated protein is tyrosine-protein kinase Blk (BLK), a Src-family kinase involved in B-cell receptor signalling. A second interesting downregulated protein is serine/threonine-protein phosphatase 2A 55 kDa regulatory subunit B delta isoform (protein 2ABD, gene *PPP2R2D*), which is the regulatory subunit of widely expressed phosphatase that plays a key in the control of mitosis entry and exit, is involved in regulation of cellular division and signal transcription and also acts as a tumour suppressor^32^. Another downregulated protein is cyclic AMP-responsive element-binding protein 1 (CREB1), which is a transcription factor that promotes the expression of inflammatory genes and has been linked with insulin resistance^33^.

In contrast, we found several significant GO terms associated with proteins that have a positive correlation between abundance and BFP (Sup. Tab. S2). Unlike in the overall GO enrichment, there was one significant term (GO:0,003,924, GTPase activity) found in the molecular function ontology. Several proteins involved in guanine nucleotide signalling show higher abundance in participants with higher BFP, amongst them RAC2, RAC1, guanine nucleotide-binding protein G(I)/G(S)/G(T) subunit beta-1 (GBB1) and guanine nucleotide-binding protein G(I)/G(S)/G(O) subunit gamma-10 (GNG10). It should be noted that RAC1 and RAC2 are additionally associated with several significant biological processes GO terms related to regulation of granulocyte/neutrophil chemotaxis, alongside chemokines such as CXCL3, which has chemotactic activity for neutrophils, and SDF1.

Prior mass spectrometry-based studies of blood plasma have shown a distinct, stable and replicated proteome profile in adult obesity/overweight^34^. After a period of weight loss, plasma levels have been reported to increase for sex-hormone binding globulin, adiponectin, and decrease for calprotectin-forming proteins S100-A8 and S100-A9, C-reactive protein (CRP) and the CD109 antigen^35^. Others studying blood proteomes have linked obesity with dysregulation of multiple molecular pathways in inflammation, cellular stress and metabolic dysregulation^36^.

Previous proteomic investigations in obesity have shown multiple, widespread and stable differences in proteomic expression between adults with overweight/obesity compared to healthy controls, especially in blood serum, adipocytes^37^ and skeletal muscles^38^. A comparison of the PBMC proteome between obese and normal weight men showed more than sevenfold increased expression of thrombospondin 1 (TSP1) and twofold decreased expression of histone deacetylase 4 (HDAC4) in the obese group, both of which were confirmed with qPCR and Western blot detection of mRNA and protein levels, respectively^39^. TSP1 is a multifunctional adipokine that plays a role in thrombosis, adipose tissue inflammation, macrophage chemotaxis and cytokine signalling^40^. While HDAC4 was not included in our set of proteins with good peptide-level evidence, we did observe increased TSP1 levels in children with higher BFP in our study, similar to what was previously shown in obese adults.

There are some clear limitations of the present study. While we show that BFP correlates with abdominal adiposity levels and can therefore be used as a readily accessible measure of body composition, it also strongly correlates with subcutaneous adiposity, which has less strong effects on metabolic health. Given our data, we cannot unambiguously distinguish how regional distributions of adipose tissue in this population affect inflammation, oxidative stress and other metabolic changes. Second, while we included leukocyte counts in the statistical model to account for the overall effect of PBMC proteome changes induced by cellular proliferation, there are likely residual effects in our dataset that we could not tease out. The interpretation of proteome profiles could be improved in a future study by separately analysing different cellular populations, although this may be difficult with limited blood sample availability, especially for rare cell populations. Another strategy that could be used is including differential blood counts instead of overall leukocyte counts, but unfortunately only overall leukocyte cell counts were available in this cohort. Finally, the generalisability of our results may be limited by features of the moderately sized cohort, such as predominantly Caucasian ethnic study population and small numbers of children with severe obesity.

In summary, the present work shows that blood proteomics unveils insight in metabolic states and inflammation processes in paediatric obesity. We showed that BFP as measured by BIA significantly correlates with PBMC expression profiles, such that the PBMC proteome is altered in children and adolescents with an unfavourable body composition, including increased expression of CUTA and several mitochondrial proteins such as NDUFS7. Furthermore, we showed that higher body fat percentage is associated with upregulation of proteins involved in leukocyte chemotaxis such as RAC2, RAC1 and THBS1. In this way the PBMC proteome reflects altered inflammation states and oxidative stress in childhood obesity.

## Methods

### Participant enrolment

Detailed information on patient enrolment and clinical data collection has been described previously^22^. Briefly, eighty participants aged 5–18 with either normal weight or overweight/obesity were enrolled in the study. For the present analysis of the PBMC proteome, one participant was excluded because a PBMC sample was not available, four were excluded because the isolated protein material was of poor quality, and four were excluded because full body composition measurements were not available, leaving seventy-one participants in the present analysis. All ethical aspects of the Helsinki Declaration were followed (World Medical Association, version October 2013). The study methods and procedures were approved by the institutional ethics committee (The Medical Ethics Committee of the University Medical Centre Maribor) and by the National Medical Ethics Committee of the Republic of Slovenia (decision 0120–78/2023/6, 16 May 2023). Informed written consent was obtained from the parents or legal guardians of participants, or directly from those aged 15 and older. All participants were informed of the voluntary nature of the study and assured of data confidentiality in line with General Data Protection Regulation and relevant national frameworks.

### Body composition

Body composition was assessed using bioelectrical impedance analysis (BIA) with the Nutrilab Bioimpedance device (Akern 2016) and Biatrodes Akern electrodes. Measurements were taken under standardized conditions, with participants fasting, having an empty bladder, and lying in a supine position. To ensure accuracy, proper electrode placement was maintained according to manufacturer’s instructions, and participants were instructed to refrain from exercise for 12 h and avoid caffeine or alcohol intake for 24 h prior to measurement. Fat mass (FM, in kg) was calculated with the manufacturer’s software (Bodygram Plus 1.2.2.8, Akern). This methodology followed the guidelines outlined in the Akern Bodygram Plus Software Guide. Additionally, abdominal ultrasound was performed by trained physicians using an abdominal transducer to measure visceral and subcutaneous fat thickness^22^. Measurements were performed according to the Ultrasound protocol for visceral fat and abdominal subcutaneous fat in both adults and young children^41^. The visceral fat thickness was defined as the distance between the peritoneum and the corpus of the lumbar vertebra, and the subcutaneous fat measurement as the distance between the skin and the ventral edge of the abdominal muscles (linea alba).

### Blood sample collection and processing

Blood samples (5 mL) were collected by trained nurses in EDTA-Vacutainers. Within one hour of collection, blood was diluted 1:1 with sterile phosphate-buffered saline solution (PBS), layered over Lympholyte-H Cell Separation Media (Cedarlane) and centrifuged at 800 rcf, 22 °C for 20 min. The buffy coat (PBMC) layer was transferred to a fresh centrifuge tube, washed with PBS and centrifuged at 800 rcf for 10 min. The resulting cell pellet was resuspended in PBS, split into three equal aliquots, pelleted and all aliquots were stored at -80 °C until further processing.

### Protein and peptide processing

Cell pellets from one PBMC aliquot were lysed in 250 μL lysis buffer (100 mM Tris·HCl, 2.5% sodium dodecyl sulfate) and gently agitated, dithiothreitol (final concentration 4 mM) and chloroacetamide (final concentration 40 mM) were added, and the protein solution reduced and denatured during incubation at 95 °C for 5 min. A portion of the lysate (80 μL) was transferred to fresh Protein Lo-Bind tubes (Eppendorf). The alkylated and reduced protein was processed following the SP4 protocol^42^: briefly, protein samples were precipitated using four volumes of acetonitrile, followed by five washes with 500 μL 80% ethanol. The protein pellet was briefly dried, then vortexed in 150 μL 100 mM TEAB buffer to facilitate resuspension. Trypsin (Trypsin Gold, Promega) was added in 1:25 m/m ratio and incubated overnight at 37 °C, 700 rpm, which facilitated full resuspension of the protein pellet. This peptide solution was clarified by centrifugation and digestion stopped with addition of 25 μL 5% formic acid. Peptides were simultaneously filtered and desalted following the R3 cleanup protocol^43^: POROS R3 cross-linked poly-divinylbenzene particles (Thermo Scientific, cat. no. 1133903) were first suspended in 96-well filter plates and conditioned by washing with 50% acetonitrile, followed by washing twice with 0.1% formic acid in water. The peptide solution was added to conditioned R3 particles, incubated for 5 min and centrifuged through the filter plate. The flow-through unbound fraction was discarded. Particle-bound peptides were washed two times with 0.1% formic acid (FA) in water and eluted in 0.1% FA, 50% acetonitrile in two portions, then evaporated to dryness.

### Liquid chromatography/tandem mass spectrometry (LC–MS/MS) data collection

Desalted peptides were resuspended in loading buffer (3% acetonitrile, 0.1% FA) and the peptide concentration was adjusted to 1.0 μg/μL. Peptides were analysed on a Ultimate 3000 RSCLnano (Thermo Scientific) HPLC system connected to a Q Exactive Plus hybrid quadrupole-Orbitrap mass spectrometer (Thermo Scientifc) set up for direct injection operation. Samples were injected in random order to minimise batch effects due to MS instrument changes. Peptides (1 μL per sample) were separated on a 25 cm long column (EasySpray, Thermo Scientific ES902; 250 mm × 75 μM internal diameter, packed with 2 μm diameter C18 particles) kept at 40 °C. The HPLC was set up with mobile phase A (0.1% FA in milliQ water) and mobile phase B (0.1% FA, 80% acetonitrile in water). The LC flow was maintained at 300 nL/min throughout with mobile phase composition changing as follows: 2 min peptide loading at 4% B, 60 min linear gradient 4%-16% B, 60 min steeper linear gradient 16%-37% B, 5 min 37%-50% B, 3 min 50%-98% B, 2 min column wash 98% B, final equilibration to 4% B. Peptides were ionised in positive ionisation mode on EasySpray source with ionisation voltage + 2.0 kV, capillary temperature 250 °C, without auxiliary or sheath gas, S-lens RF level 50. Mass calibration was checked twice per week and recalibrated as needed.

Q Exactive Plus spectrometer was operated in Top14 data dependent mode, switching automatically between MS and MS/MS acquisition. Full scan MS spectra (m/z 200–2000) were acquired with a mass resolution 70 000, AGC target 3e6, maximum injection time 100 ms, followed by up to 14 sequential high-energy collisional dissociation MS/MS scans with a resolution of 35 000 (NCE 27, isolation window 2.0 m/z, AGC target 1e5, maximum injection time 50 ms). Ions with charges other than 2, 3 or 4 were excluded from MS2 collection. All data were collected in centroid mode. Data acquisition was completed within two weeks, and LC–MS/MS instrument performance was monitored by periodic standard acquisition.

### Database searching and preprocessing

Protein identification and quantitation was performed in Proteome Discoverer (v2.4.1.15 SP1, Thermo Scientific) in a two-step workflow. The first step was adapted from Precursor Quan and Sequest HT Percolator default workflow. Each raw file was searched with Sequest HT against the reviewed SwissProt human proteome (Uniprot 9606, 20 342 sequences, downloaded 29 Sep 2023). The Hao group list of common contaminants was added as a second database^44^. Tryptic peptides only were allowed (up to 2 missed cleavages, peptide length 6–40 amino acid residues) with precursor mass tolerance set to 10 ppm and fragment mass tolerance to 0.02 Da. Cysteine carbamidomethylation was set as a global fixed modification, methionine oxidation as global dynamic modification, and acetylation and/or methionine loss as dynamic modification at protein N-terminus. Peptide spectrum matches were evaluated with Percolator against a concatenated target/decoy database with default settings (workflow shown in Sup. Fig. S6). Resulting MSF files were used in a consensus analysis (workflow shown in Sup. Fig. S7) with the following options: features were aligned with maximum retention time shift set at 20 min; precursors were quantified based on intensity and no normalisation or scaling was performed at this stage.

Peptides, proteins and input file tables were exported from consensus analysis results and used for further analysis in R Statistical Software (v.4.3.0, https://www.r-project.org/ ^45^ The workflow was adapted from a previously published process^46^ which builds on the QFeatures package (v.1.12.0, https://bioconductor.org/packages/QFeatures) for proteomics data handling^47^. After initial raw sample quality assessment, contaminant peptides, peptides without protein accessions, peptides without reliable quantity data, peptides belonging to multiple protein groups and ambiguous features were filtered out. Protein tables were used to remove peptides belonging to proteins that exceed dataset-wide FDR cutoffs (0.01) at peptide or protein level, based on a target-decoy strategy. Additionally, peptides present in < 10% of samples were removed. Peptide intensities were log2 transformed, aggregated to proteins based on master protein accession using robustSummary aggregation (MS core utils, v1.14.1,

https://rdrr.io/bioc/MsCoreUtils/) and protein intensities normalised using “diff.median” method. We excluded proteins that were only supported by a single peptide, since these are more likely to have unreliable quantification. Only proteins present in ≥ 50% samples were used for further analysis, which excludes proteins identified in only a small subset of samples due to inherent stochastic effects in data-dependent acquisition. In total we identified a total of 49 082 peptides passing quality filters, of which 38 324 were present per sample on average (Sup. Figs.  S8, S9, S10).

### Statistics

 Differential protein abundance according to BFP and leukocyte count was explored with moderated *t*-tests, adjusted for participant sex, protein isolation batch and MS analysis date, implemented in the limma R package (v.3.58.1, https://bioinf.wehi.edu.au/limma/)^48^ Limma moderated *t*-tests employ an empirical Bayes method to borrow information on protein variance across all proteins in the experiment, which increases the power while remaining robust even with small sample numbers. Covariates were selected based on observed correlations in clinical parameters (sex, leukocytes) and technical factors that are known to cause batch effects in shotgut proteomic analysis (isolation batch, analysis date). Differential expression was considered for proteins with q-value < 0.05 regardless of the magnitude of correlation. Gene Ontology analysis was done with clusterProfiler R package (v.4.10.0, https://bioconductor.org/packages/release/bioc/html/clusterProfiler.html)^49^.

## Supplementary Information

Supplementary Information 1.

Supplementary Information 2.

## Data Availability

The mass spectrometry proteomics data have been deposited to the ProteomeXchange Consortium via the PRIDE partner repository with the data set identifier PXD064634 .
