# Supplementary material for: PBMC proteome is altered in children with high body fat percentage
Source: Sci Rep. 2025 Nov 19;15:40733. doi: 10.1038/s41598-025-24461-2 (PMC12630839; doi:10.1038/s41598-025-24461-2)
Supplement: Supplementary file 1 — Supplementary Information 1. [file 41598_2025_24461_MOESM1_ESM.docx]

## Supplemental Information

## Supplemental Figures


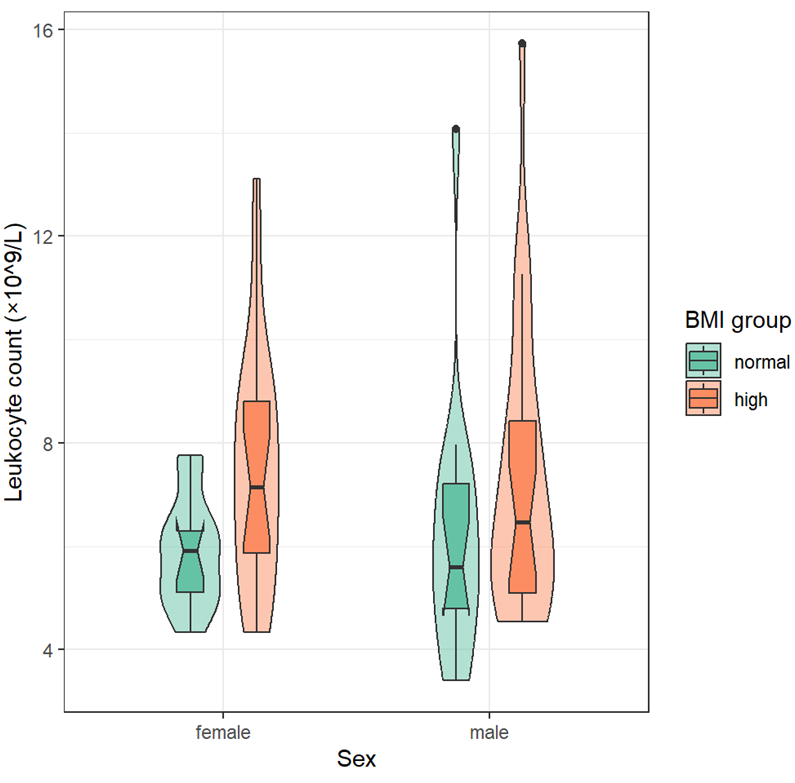


**Sup. Fig 1**: Distribution of leukocyte counts in male and female participants. Boxplots first quartile (lower line), median (middle thick line) and third quartile (upper horizontal line) in each group, with whiskers extending to the smallest and largest values no further than 1.5× interquartile range away. Notches around the median show the 95% confidence interval for each group median.


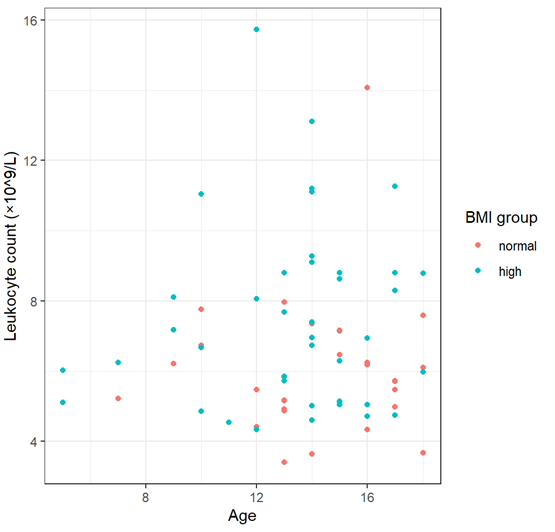


**Sup. Fig 2**: Changes in the leukocyte count (A) and in PBF (B) as a function of age. Participants with BMI in the normal weight range are represented by blue dots and participants with overweight or obese BMI values are shown in red. Blood leukocytes count normally decreases from infancy throughout childhood and adolescence.


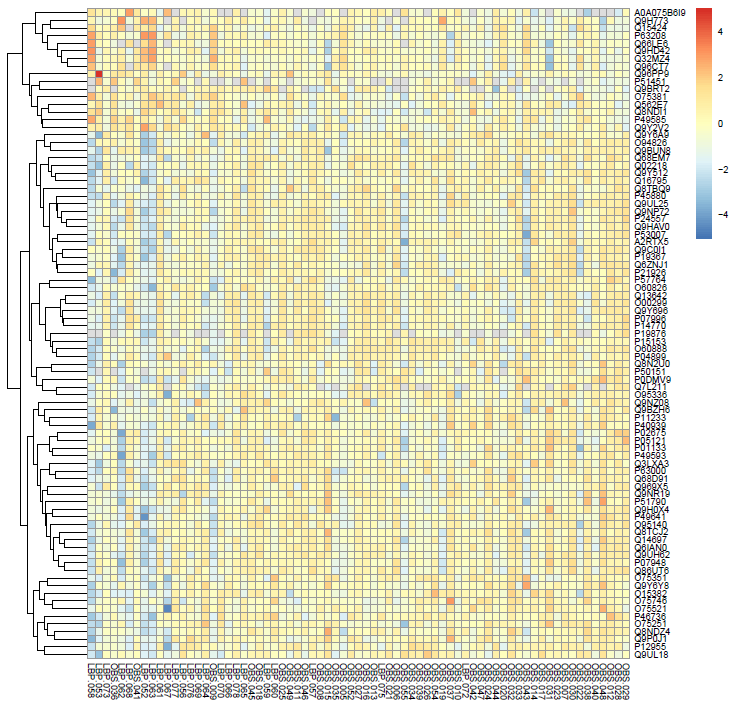


**Sup. Fig. 3**: Heatmap of protein abundance changes for 85 significantly abundant proteins across all patients. Each column represents a patient, ordered left to right by increasing PBF. Each row represents a protein with protein Uniprot accession displayed on the right-hand side. All protein abundances are scaled to the same average to enable comparison between proteins.


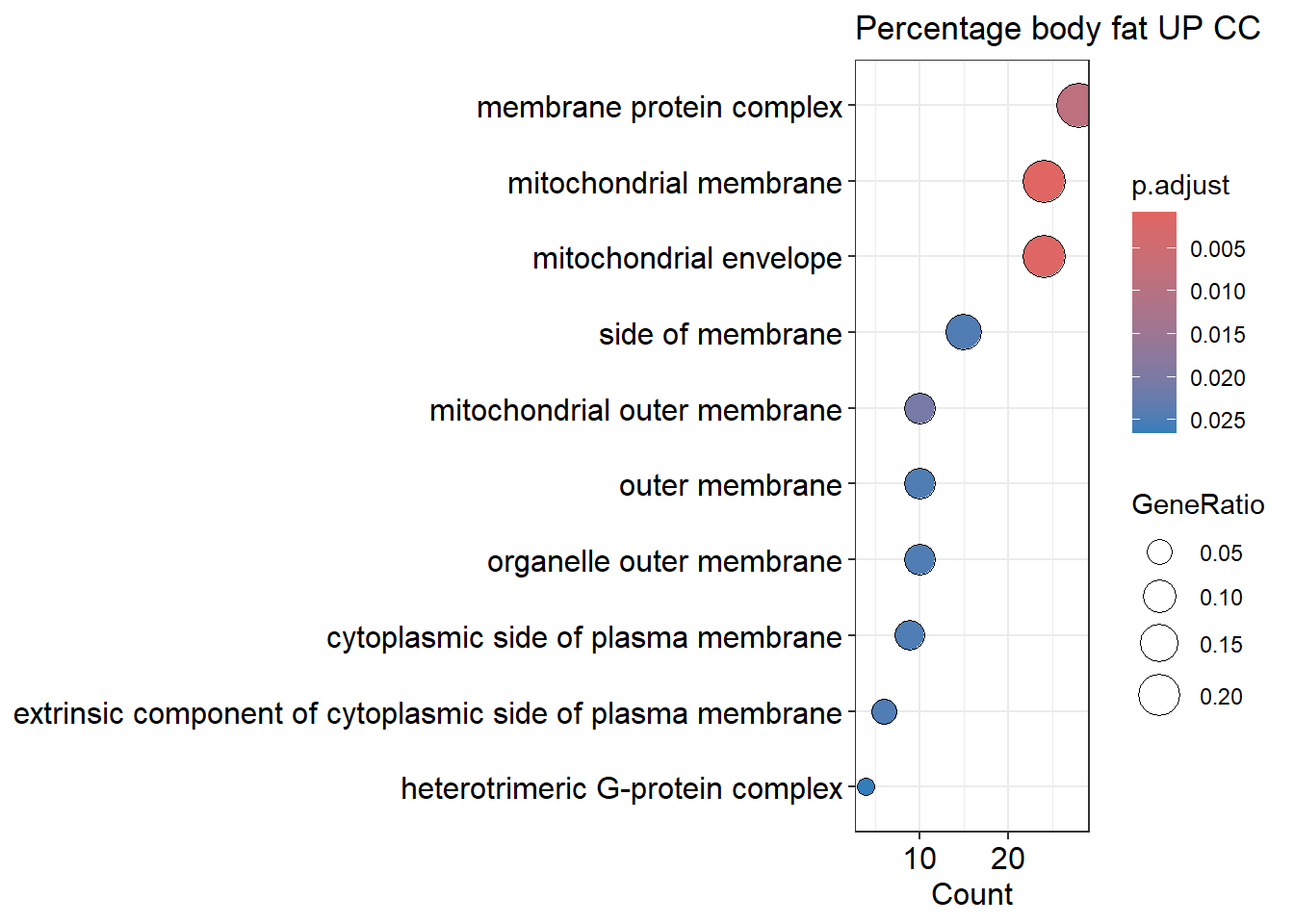


**Sup. Fig. 4**: Cellular component GO enrichment for upregulated proteins in associating with BFP. Count indicates the number of protein accessions with the GO term and p.adjust the adjusted P value for GO enrichment.


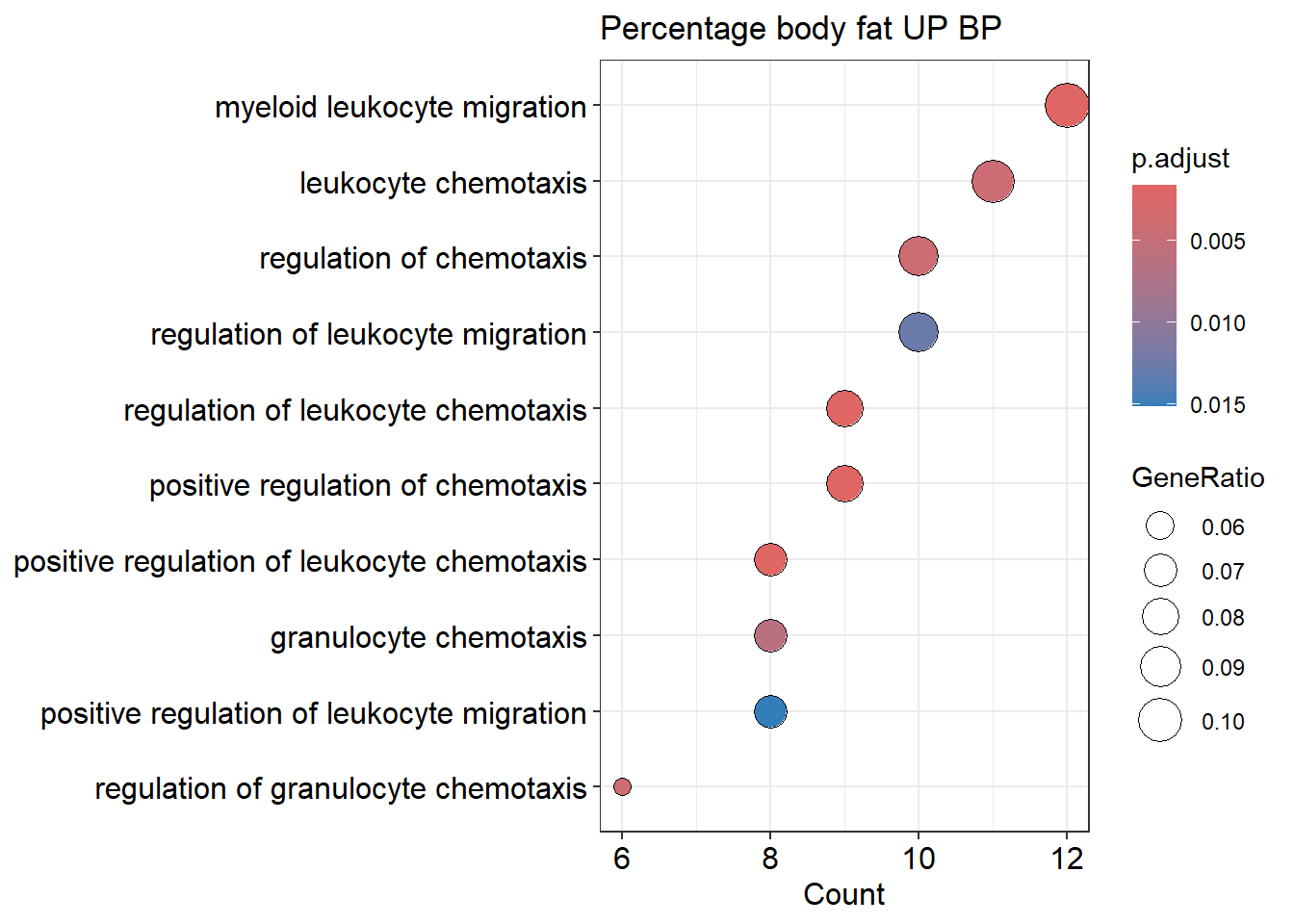


**Sup. Fig. 5**: Biological processes Gene Ontology enrichment of significant terms for proteins with higher abundance associated with higher BFP. Count indicates the number of protein accessions with the GO term and p.adjust the adjusted P value for GO enrichment.


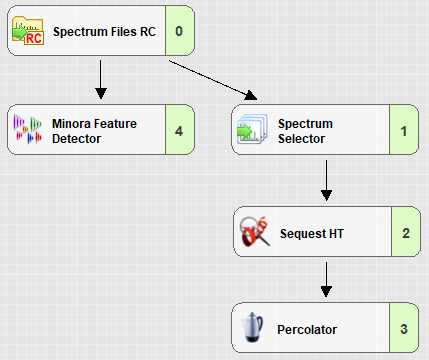


**Sup. Fig 6**: Proteome Discoverer processing workflow, adapted from PWF_QE_Precursor_Quan_and_LFQ_SequestHT_Percolator with modifications.


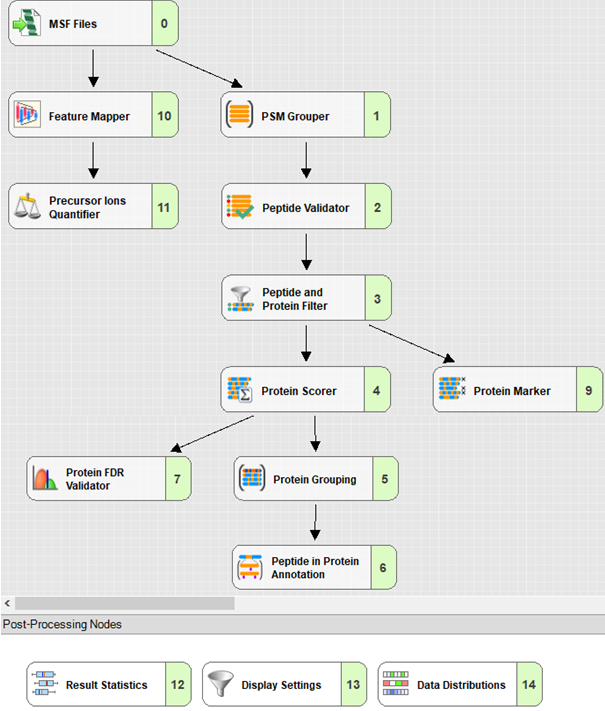


**Sup. Fig 7**: Proteome Discovere consensus workflow nodes. Adapted from CWF_Comprehensive_Enhanced Annotation_LFQ_and_Precursor_Quan with modifications for peptide databases, absence of normalisation and contaminant protein marking.


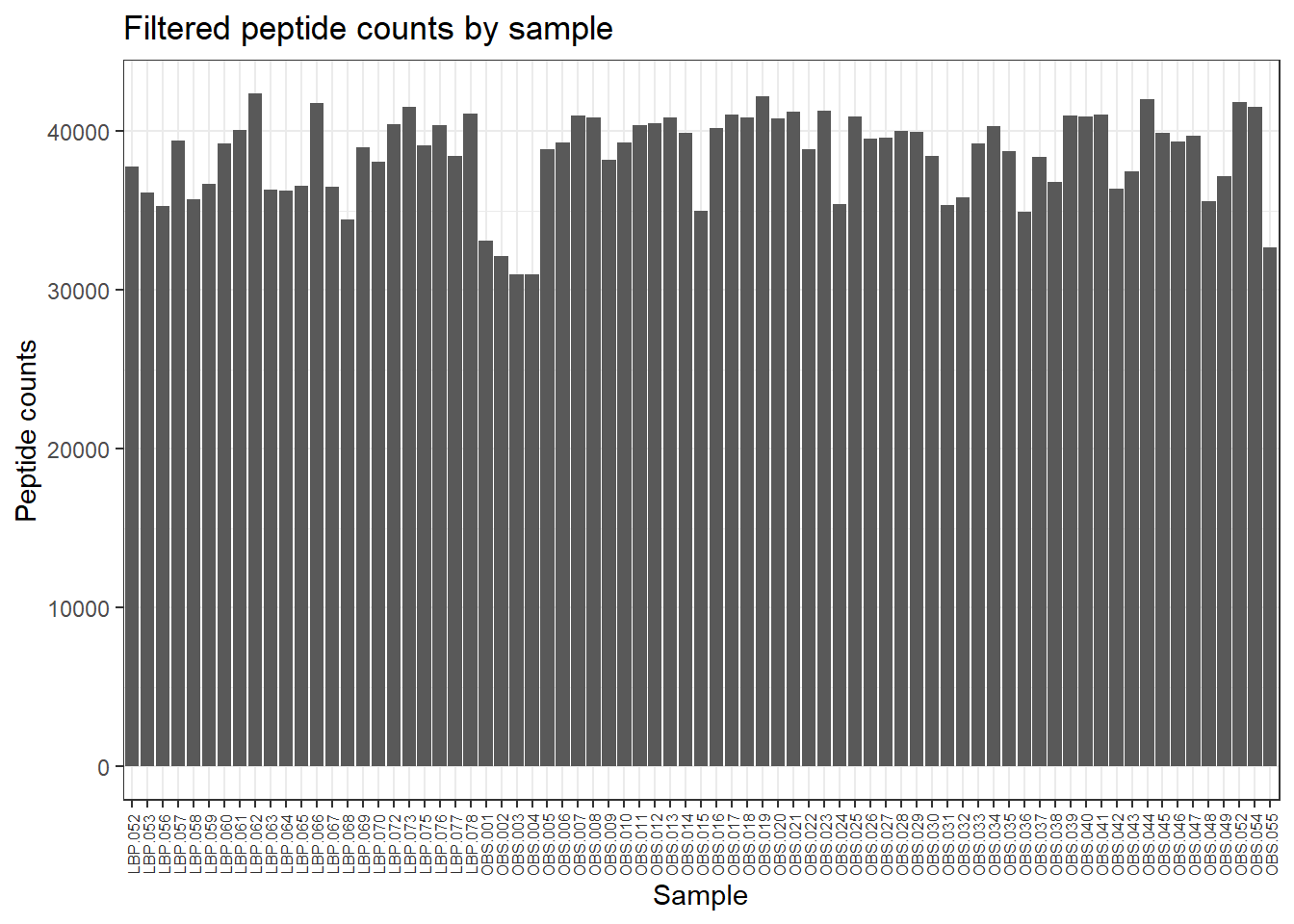


**Sup. Fig 8**: Number of quantified peptides per sample after quality filters.


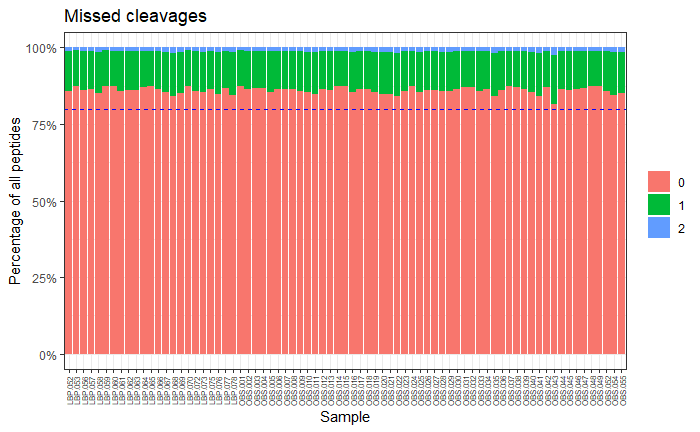


**Sup. Fig 9**: Missed cleavages in identified peptides by sample. Horizontal dotted line indicates 80%, a quality threshold for acceptable rates of missed cleavages.


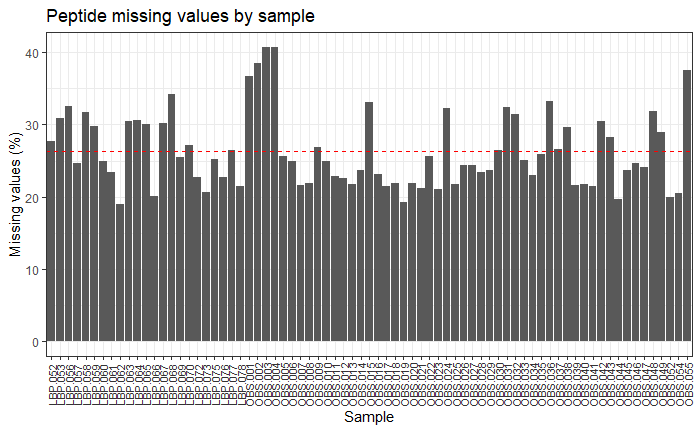


**Sup. Fig 10**: Data completeness for peptide intensities in peptide passing quality filters. Red dotted line indicates average percentage of missing values across the dataset.

## Supplemental Table Legends

**Supplementary Table S1**: Results of differential abundance comparison by body fat percentage for all 4068 proteins in the study, calculated by master protein accession in each protein group. LogFC, log2-fold change in protein abundance per % change in BFP, i.e. the slope of the regression coefficient. Average expression is given after dataset normalisation on logarithmic scale. Gene names were derived from protein accessions by lookup on Uniprot.

**Supplementary Table S2**: Significant GO terms in gene set enrichment analysis for upregulated proteins, reported for all three ontologies.
